# Chronotherapy of Non-Steroidal Anti-Inflammatory Drugs May Enhance Postoperative Recovery

**DOI:** 10.1038/s41598-019-57215-y

**Published:** 2020-01-16

**Authors:** H. Al-Waeli, B. Nicolau, L. Stone, L. Abu Nada, Q. Gao, MN. Abdallah, E. Abdulkader, M. Suzuki, A. Mansour, A. Al Subaie, F. Tamimi

**Affiliations:** 1 Faculty of Dentistry, McGill University, 2001 Avenue McGill College Suite 500, Montréal, QC H3A 1G1 Canada; 2 Faculty of Dentistry, McGill University, Strathcona Anatomy and Dentistry Building, Montreal, QC H3A 0C7 Canada; 3 Faculty of Dentistry, University of Toronto, 124 Edward St, Toronto, Ontario M5G 1G Canada

**Keywords:** Predictive markers, Medical research

## Abstract

Postoperative pain relief is crucial for full recovery. With the ongoing opioid epidemic and the insufficient effect of acetaminophen on severe pain; non-steroidal anti-inflammatory drugs (NSAIDs) are heavily used to alleviate this pain. However, NSAIDs are known to inhibit postoperative healing of connective tissues by inhibiting prostaglandin signaling. Pain intensity, inflammatory mediators associated with wound healing and the pharmacological action of NSAIDs vary throughout the day due to the circadian rhythm regulated by the clock genes. According to this rhythm, most of wound healing mediators and connective tissue formation occurs during the resting phase, while pain, inflammation and tissue resorption occur during the active period of the day. Here we show, in a murine tibia fracture surgical model, that NSAIDs are most effective in managing postoperative pain, healing and recovery when drug administration is limited to the active phase of the circadian rhythm. Limiting NSAID treatment to the active phase of the circadian rhythm resulted in overexpression of circadian clock genes, such as Period 2 (Per2) at the healing callus, and increased serum levels of anti-inflammatory cytokines interleukin-13 (IL-13), interleukin-4 (IL-4) and vascular endothelial growth factor. By contrast, NSAID administration during the resting phase resulted in severe bone healing impairment.

## Introduction

Postoperative recovery following invasive surgical interventions is usually painful and can cause significant morbidity and even mortality in some cases^1^. Postoperative pain is a response to injury in which inflammatory mediators are released, inflammatory cells infiltrate the damage site, and nociceptive nerve fibres are activated to produce pain^2^. Pain relief is crucial for full recovery; it is essential for the healing process and to resume physical activities^3^. However, drugs typically used for postoperative pain management are problematic. Acetaminophen is ineffective in severe pain, and while NSAID and opioids are useful for controlling surgical pain^4^, opioids can cause constipation and addiction, and NSAID can delay healing^5–8^. Therefore, there is an urgent need for better strategies to manage postoperative pain. One way of achieving this is by developing NSAID treatments that control pain and inhibit inflammatory catabolic activities while sparing the anabolic pathways of wound healing.

Inflammation is essential in healing^9^. For example, when connective tissues are injured, an inflammatory response starts by the conversion of arachidonic acid (AA), either into prostaglandin H2 (PGH2) via cyclooxygenase (COX), or into interleukotrien A4 (LTA4) via 5-lipoxygenase (5-LO). Downstream specific synthetase enzymes convert PGH2 and LTA4 into bioactive lipid mediators, such as prostaglandin E2 (PGE2), which play a crucial role in tissue repair^9,10^. Also, release of pro-inflammatory cytokines and growth factors at the injury site, results in increased secretion of prostaglandins. Prostaglandins contribute to the regulation of mesenchymal cells^11^, thus by inhibiting the production of PGE2 via COX, NSAIDs reduce the production of PGE2, and subsequently inhibit the healing of conective tissues (bone, cartillage, dermis, etc) (Table S1).

**Figure 1:**
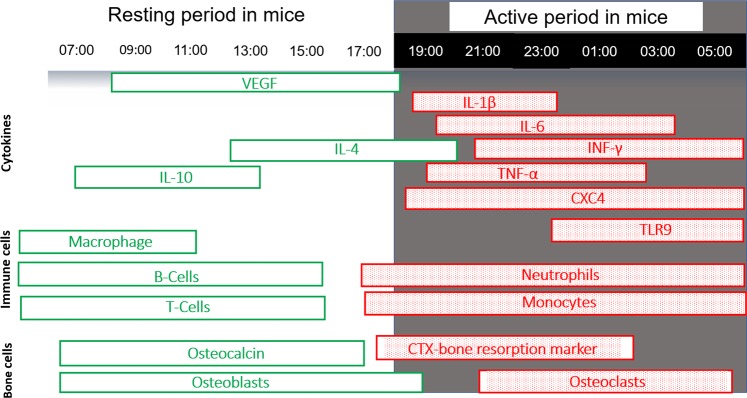
Diagram of the circadian rhythm in immune cells, pro-inflammatory and anti-inflammatory mediators, bone resorption and formation markers.

All living organisms possess a circadian rhythm that anticipates the response to changes during the 24-hour cycle^12^. The circadian system in mammals is composed of a central clock within the suprachiasmatic nuclei and peripheral clocks inside all cells. The circadian clock is controlled through a feedback loop of heterodimer core clock genes composed of circadian locomotor output cycles kaput(Clock) and brain and muscle Arnt such as protein-1(Bmal1). Clock and Bmal1 drive the expression of two inhibitors, cryptochrome(Cry) and period(Per)^13^. This molecular clock modulates the immune response and the healing processes in connective tissues^13^ (Fig. 1 and Table S2). For instance, macrophage activity, leukocyte recruitment, and pro-inflammatory mediators such as interleukin-1β (IL-1β), interleukin-6 (IL-6), and interleukin-12 (IL-12) increase at the beginning of daily activity. During this phase, the levels of Tol-Like Receptors TLR9 and TLR4 also increase, leading to the upregulation of CCL2, CXCL1, CCL5, and subsequent leukocyte recruitment and potential tissue damage in injured sites^12–15^(Fig. 1). By contrast, anti-inflammatory mediators and other growth or angiogenesis factors, such as the vascular endothelial growth factor (VEGF), peak during the resting phase^13,16,17^ (Fig. 1 and Table S2).

The circadian rhythm affects many aspects of connective tissue metabolism^18^. A 24-hour oscillation occurs in bone tissue during growth^19^, formation, resorption^20,21^, and in the endochondral ossification during bone fracture healing^21^. Bone formation occurs during the resting period, and resorption occurs mostly during the active period^21^. Experimental studies in rodents and humans reveal that the disruption of sleep and circadian rhythm impairs bone formation^22^. All bone cells such as osteoblasts, osteoclasts, and chondrocytes express clock genes, such as Per or Cry, that influence bone volume regulation^23,24^. Cry2 influences the osteoclastic activity and Per2 regulates osteoblast activity^25^. The circadian clock also affects pain, with sensitivity peaking during the active phase^26^. Part of the pain response oscillation could be explained by changes in COX-1 and COX-2 activity throughout the day^27^, especially after an injury or insult^28^. These variations may contribute to the clinically evident circadian variations in the pharmacokinetics effects of NSAID. Specifically, maximum absorption and effectiveness are achieved when the drug is administered during the active phase when animals are awake^29–33^.

Different clinical studies suggest that careful selection of the time of administration can improve the effectiveness of NSAID and can markedly reduce their undesirable effects^34^. These drugs exert a strong anti-inflammatory effect when ingested or injected in the morning or early afternoon, but not in the evening, when the risk of its adverse effects such as indigestion, stomach ulcers, and acute kidney problems increase^35^.

In this study, we investigated how time of NSAID administration impacts postoperative pain and healing. We **hypothesized** that circadian variations in inflammation caused by clock genes could determine the postoperative effectiveness of NSAID therapy. Accordingly, limiting NSAID administration to the beginning of the daily activity phase should improve recovery. To test our hypothesis, we assessed the effect of surgery time and time of NSAID delivery on pain and healing outcomes in a bone fracture surgical model in mice. We determined the most efficient delivery time for NSAID after a bone fracture surgery. Moreover, we investigated the molecular differences between the healing sites of mice that received NSAID during the active phase and those that received it during the resting phase by characterizing the gene expression profile of fracture calluses in both groups.

## Results

### The timing of bone fracture does not affect bone healing and recovery

**Figure 2:**
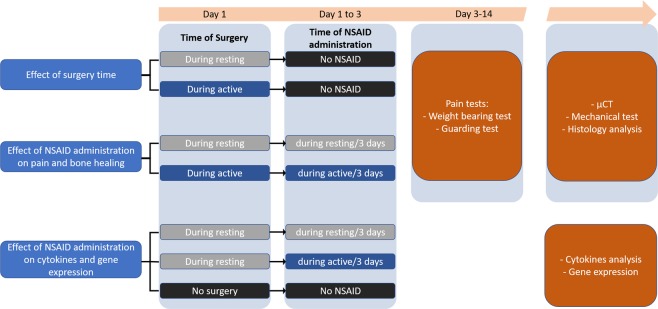
Study design and objectives.

To set the basis for the assessment of NSAID chronotherapy, we first investigated the effect timing of bone fracture on bone healing and recovery. We carried out this experiment by comparing pain behaviour and bone healing of mice that received tibia fracture surgery during the resting phase (when animals are sleeping) to those that received the fracture during the active phase (when they are awake) (Fig. 2).

**Figure 3:**
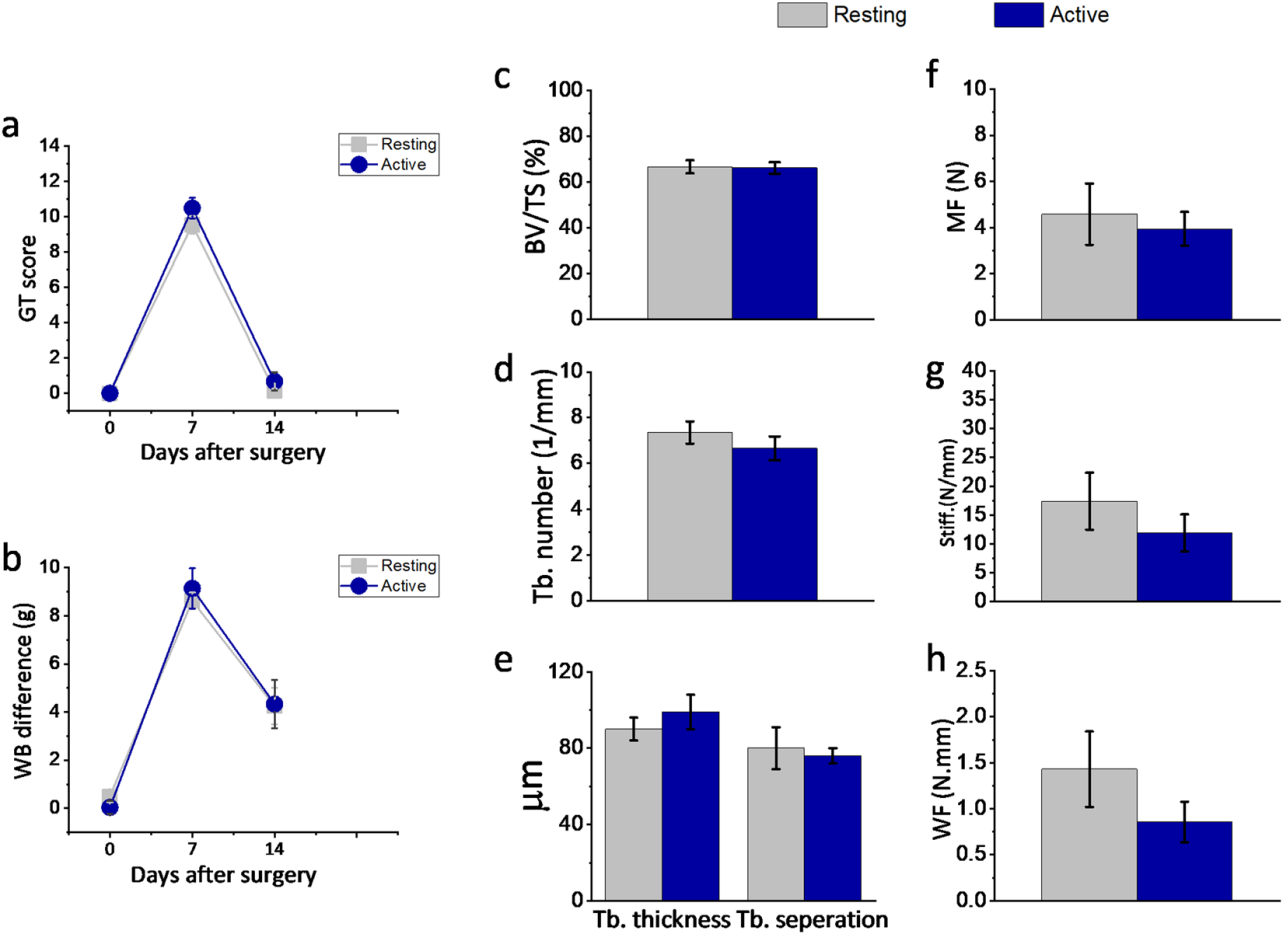
Effect of bone fracture surgery timing on pain and bone healing. (**a**,**b**) Pain assessment, (**c**–**e**) μCT analysis and (**f**–**h**) mechanical analysis of tibiae fracture during resting time and active time at day 14 after tibia fracture surgery. The data show that both groups (n = 8 per group) had similar pain behaviour (weight bearing (WB), guarding tests (GT)), bone volume fraction (BV/TV), trabecular number (Tb.N), spacing between the trabecula (Tb.Sp), trabecular thickness (Tb.Th), maximum force (MF), stiffness (S) and work to failure (WF). All data are expressed as Mean ± SE values.

There were no significant differences in pain behaviour between mice that received tibia surgery during the active or resting phases (Fig. 3a,b). The guarding of the injured limb increased, but it returned to almost pre-fracture levels by day 14. Similarly, the tendency to bear more weight on the uninjured versus the injured limb increased after the bone fracture, but partially resolved by day 7.

Two weeks following surgery, the osteotomy sites of the fractured tibiae were subjected to micro computed tomography (µ-CT) analyses to assess bone healing (Fig. 3c,d,e). We did not observed significant differences in the bone volume to tissue volume (BV/TV), callus size, trabecular number (Tb.N), or spacing between the two experimental groups. Moreover, the biomechanical analysis revealed no significant difference in the force to fracture, stiffness or work to failure measurements between the bones harvested from the two experimental groups (Fig. 3f,g,h).

### The timing of NSAID administration affects bone fracture pain and recovery

To assess the effect of the timing of NSAID administration on bone fracture pain and recovery, we used a methodology like that discussed in the previous experiment (Fig. 2). We evaluated the pain behaviour of two groups of mice following a tibial fracture. For pain management, after the bone fracture, one group received NSAID at the beginning of the active phase, and the other group received NSAID at the end of the active phase.

**Figure 4:**
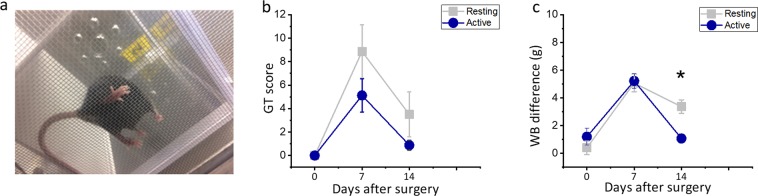
Effect of NSAID dose timing on pain behaviour. Effect of NSAID administration on pain behaviour (n = 8 per group) at 0, 7 and 14 days after surgery. (**a**) Guarding test mesh for the paw of the fractured leg, (**b**) guarding test, (**c**) weight bearing test. Scores are expressed as mean ± SE values *P < 0.05.

Immediately after inducing a tibial fracture, all mice increased limb guarding and decreased weight bearing on the injured limb, which are both indicators of pain. Two weeks after surgery, the mice in the group that received NSAID during the active phase recovered the weight bearing in comparison to pre surgery values (P = 0.85, Fig. 4c). However, mice treated during the resting phase still showed slower recovery after two weeks in comparison to pre surgery scores in guarding and weight bearing tests (p < 0.05, Fig. 4b,c). Also, the mice treated during the active phase bore significantly more weight on the injured limb at day 14 compared to the resting phase group (p < 0.05, Fig. 4c).

**Figure 5:**
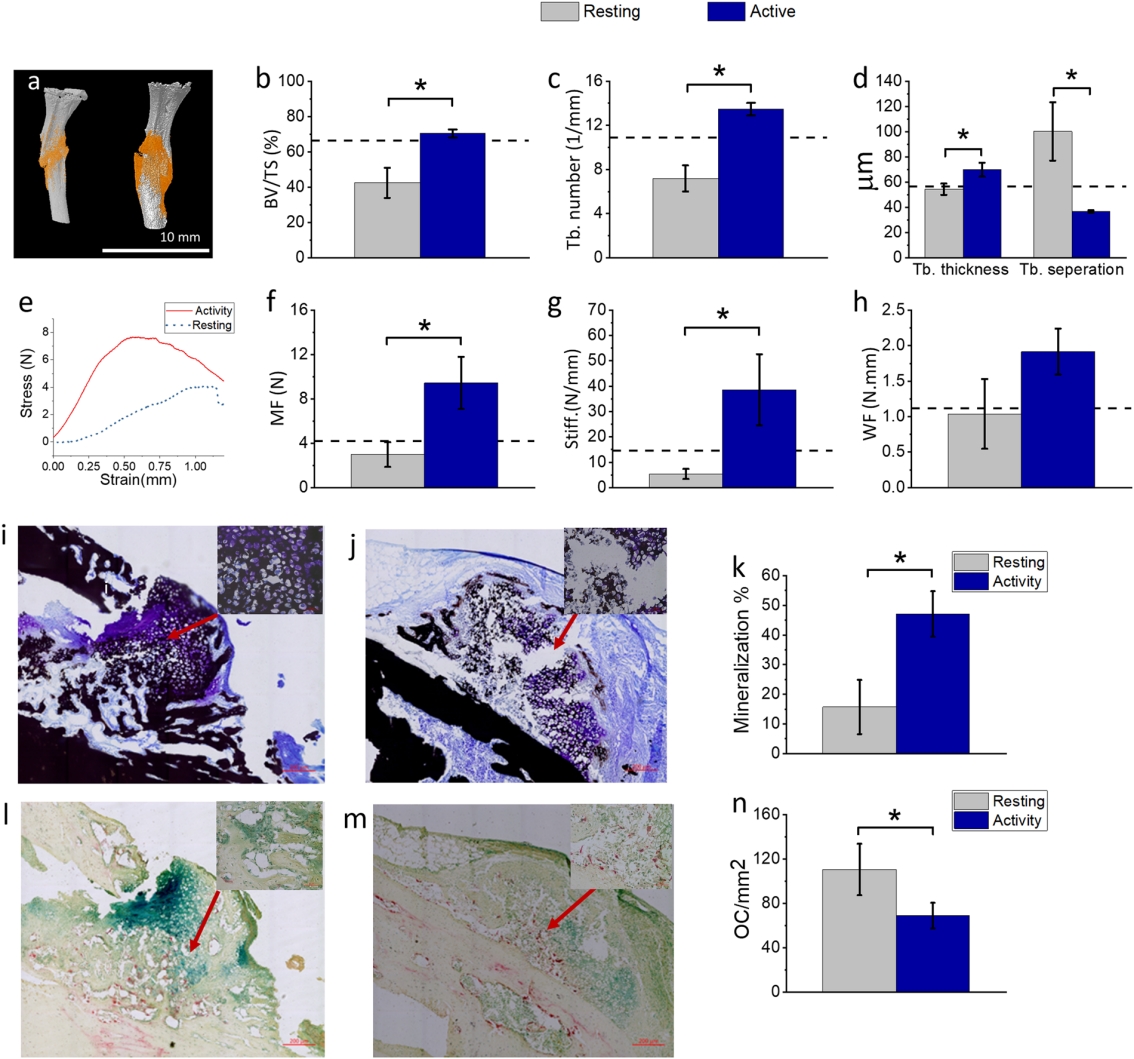
Effect of NSAID dose timing on bone healing outcomes. (**a**) μCT images of tibiae retrieved from the NSAID resting time group (left) and NSAID active time group (right) at day 14 after tibia fracture surgery, dotted line represents the average results without NSAID administration from Fig. 3. The group that received NSAID at the active time had (**b**) a higher bone volume fraction (BV/TV) and (**c**) trabecular number (Tb.N), (**d**) lower spacing between the trabecula (Tb.Sp) and higher trabecular thickness (Tb.Th) compared to the group receiving NSAID at resting time (n = 7 per group). (**e**) The mechanical stress-strain curve of 3-point bending tests on the fractured bones, (**f**) maximum force (MF), (**g**) stiffness (Stiff.) and (**h**) work to failure (WF) of the fractured tibiae of each group (n = 7 per group), dotted line represent the average results without NSAID administration from the surgery time experiment (Fig. 3). (**i**–**n**) Histology Von-Kossa-stained sections: (**i,j**) mineralization in black at fracture sites in mice (**i**) receiving NSAID during resting time or (**j**) during active time. (**k**) Percentage of mineralized tissue within the callus. Tartrate-resistant acid phosphatase (TRAP) stain (**l**,**m**) shows the osteoclasts in the fracture site of mice receiving NSAID (**l**) during resting time and (**m**) active time (scale bars represents 200 μm (big image, and 10 μm small image)). (**n**) Number of osteoclasts per unit area of callus (n = 4 per group). All data are expressed as Mean ± SE values. *P < 0.05.

To assess the effect of NSAID timing on bone healing, we analyzed the morphology and mechanical properties of the harvested bones from the mice of the two groups described above. Two weeks after inducing the bone fracture, µ CT analyses revealed that NSAID treatment during the active phase resulted in significantly larger callus at the fracture sites, and higher bone volume to tissue volume, trabecular number, and decreased spacing between the trabeculae, than NSAID treatment during the resting phase (Fig. 5b,c,d) (p < 0.05). Histomorphometric analysis of the fractured bones also revealed a significantly larger callus area and greater mineralized bone area in mice treated during the active phase compared to the resting phase (Fig. 5k). This was associated with significantly fewer tartrate-resistant acid phosphatase (TRAP)-stained osteoclasts in the animals receiving NSAID during the active phase (Fig. 5n).

To investigate whether differences in biomechanical properties accompanied the difference in the structure of the healing callus, the biomechanical characteristics of the healing bones from the two experimental groups were investigated by three-point-bending tests (Fig. 5e,f,g,h). Analysis of the load-displacement curves showed higher maximum force to fracture and stiffness in the fractured bones of the animals treated during the active phase, in comparison to the mice receiving treatment during the resting phase (Fig. 5f,g,h). It is interesting to observe that the administration of NSAID at the resting time resulted in impaired healing compared to mice not treated with NSAID, whereas the “active time “treatment had the opposite effect.

Due to the importance of our results and their potential clinical implications, we decided to repeat part of the experiments to confirm the results. This was done by a different group of researchers (QG MS) that repeated independently part of the experiments in order to further validate of our results. This included the experiments designed to assess the effect of surgery time, and the timing of NSAID administration after bone fracture, and the subsequent analyses (pain and behavioural tests and bone healing assessment through micro CT scan and mechanical bending tests).

### The timing of NSAID administration affects systemic inflammation after bone fracture

**Figure 6:**
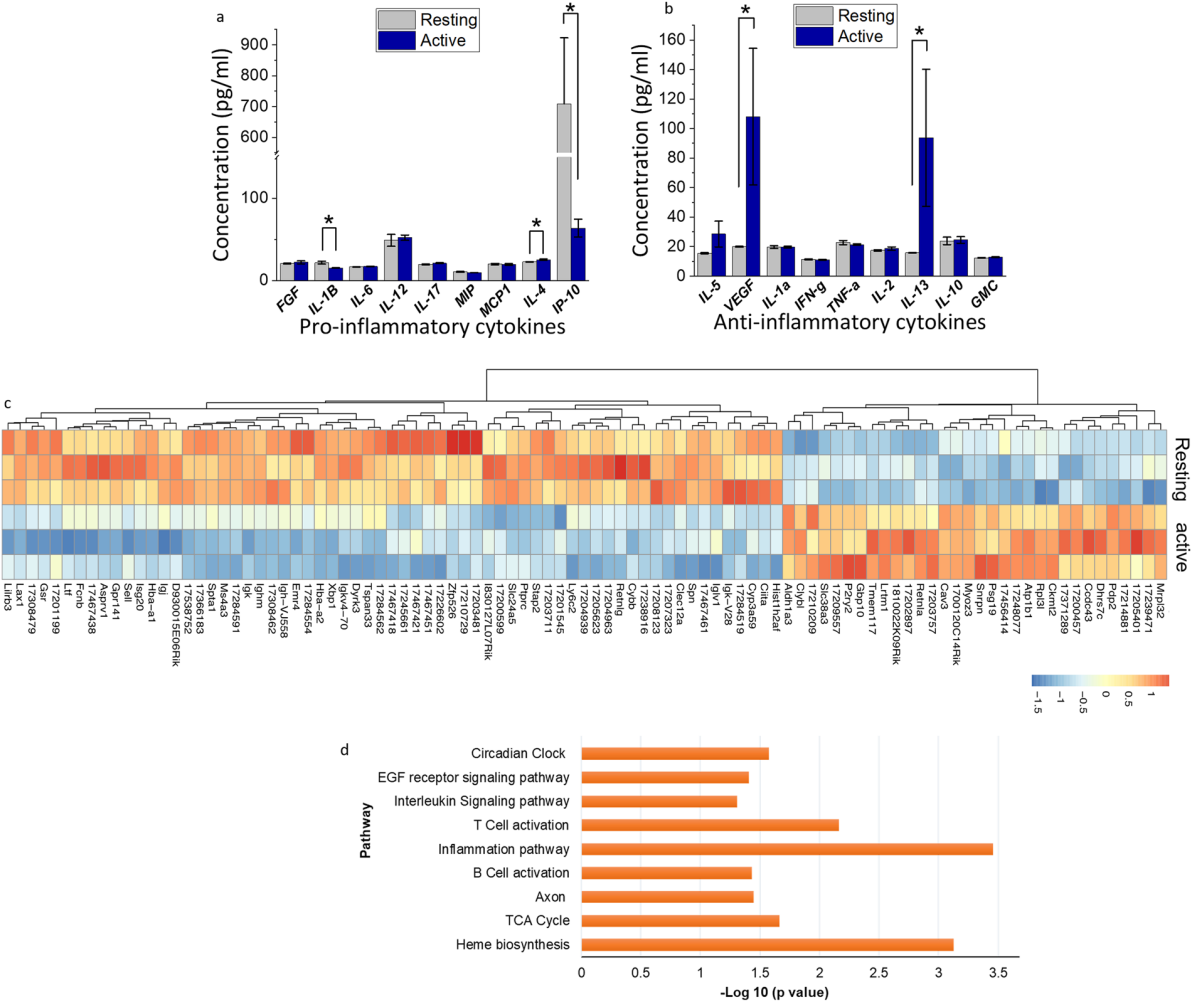
Effect of NSAID dose timing on systemic inflammatory cytokines and gene expression of the healing callus. (**a**,**b**) The concentration of serum cytokines during fracture healing: (**a**) serum concentrations of pro-inflammatory cytokines, and (**b**) anti-inflammatory cytokines and growth factors. (**c**) Gene expression heat map for the top 100 genes that showed at least 1.5-fold difference (P < 0.05), upregulated (red) or downregulated (blue), at day three after fracture surgery for the group receiving NSAID at active time compared to those receiving NSAID at resting time. (**d**) PANTHER pathway ontology analysis showing significantly enriched Panther pathways (p < 0.05) of differently expressed genes between the group that received NSAID at the active time compared to resting time at day 3 after fracture surgery. Concentrations are expressed as mean ± SE values. *P < 0.05.

To understand how the timing of NSAID administration after bone fracture affects the systemic inflammatory response, we measured the levels of 20 inflammatory cytokines in serum. These analyses revealed increased levels of interleukin IL-13, vascular endothelial growth factor, and IL-4, and decreased levels of IL-1β and interferon gamma-induced protein 10 in the mice receiving treatment during the active phase compared to those receiving it during the resting phase (p < 0.05) (Fig. 6a,b).

### The timing of NSAID administration affects gene expression in bone fractures

The effect of the timing of NSAID administration on the expression of genes at the bone fracture site was assessed using RNA microarray. Extracted total RNA pools were harvested on day 3 after surgery and examined for global gene expression profiles (Figs. 6c,d and S3a–c and Tables S3 and S4). NSAID administration during the active phase elicited a different gene expression profile in comparison to the resting phase group. In comparison to the negative control group (no NSAID and no surgery), there were 8300 genes expressed in the group that received NSAID during the active phase after bone fracture, and 7200 genes expressed in the group that received NSAID during resting time after bone fracture (Fig. S3a,b) (Data Set-Series GSE126648-GEO Repository). In total, 555 genes exhibited a significant difference in expression of ≥1.5-folds (p < 0.05) between the two groups (Fig. 6c), including specific genes involved in the bone healing process and macrophage polarisation, such as CCl12, Retnla, CCl3 and FGF growth. In addition, clock genes such as Per2 were upregulated by NSAID administration during the active phase. Moreover, genes known to impair bone healing, such as STAT1, were downregulated in the active phase group (Table S4).

According to a ‘gene ontology’ classification, the genes affected by the timing of NSAID administration were mainly related to cellular, metabolic and biological regulation processes (Table S3 and Fig. S1a,b). Also, they were associated with nine different signaling pathways, such as the circadian clock, inflammation mediated chemokines and cytokines, epidermal growth factor receptor (EGFR) pathway, interleukin, T cell activation, B cell activation, axon, TCA cycle, and heme biosynthesis signaling pathways (Fig. 6d). Among these pathways, the circadian clock and EGFR signaling pathways were expressed in the group that received NSAID during the active phase but not during the resting phase.

## Discussion

Our results suggest that NSAID administration during the daily activity period results in better postoperative healing and recovery in a bone surgery model. This is likely due to the impact of NSAID timing on inflammation at the healing site, especially through the overexpression of circadian clock genes such as Per2. This effect of NSAID chronotherapy seems to be independent of the time of surgery.

The expression of the genes involved in the circadian clock pathway and circadian rhythm biological process was affected by NSAID chronotherapy (Fig. 6d). Clock genes such as Per2, Nr1d1, and Nr1d2 appeared to be upregulated in the fracture sites of the group that received NSAID during the active phase, in comparison to the resting phase group. This effect of NSAID chronotherapy is probably through the COX-independent pathway. NSAID administration has been shown to affect Per2 clock gene expression in canine cancer cell lines^36^. Other studies have shown that independent of COX pathways, NSAID administration affects a variety of transcription factors that regulate Per2 clock gene expression, such as EGR-1^37,38^, B- Catenin/TCF^39^, NF-кB^40^, ATF3 and ATF4^41^. Some of these transcription factors were expressed in both experimental groups in comparison to the control group.

There is an increasing interest in the possibility of targeting of the circadian clock in therapeutic approaches to control inflammatory diseases, metabolic diseases, and cancer; in addition to promoting healing and maintaining hemostasis^42,43^. Our gene ontology analysis of the differentially expressed genes suggested that the timing of NSAID administration after bone fracture affected several signalling pathways such as: the inflammation mediated pathway, the interleukin, T-cell and B-cell activation signalling pathways, and the EGFR signaling pathway. Studies have demonstrated the effect of these pathways^44–46^ on regulating cytokine signaling and the immune response.

RNA microarrays of the fracture site among the group that received NSAID during the active time revealed an upregulation of genes signalling cytokines associated with the polarization of macrophage cells from M1 phenotype to M2 such as Retnla, FIZZ1 and IL-4R1 during bone healing^47^. This group also showed expression of genes associated with the recruitment of mesenchymal stem cells and the initiation of the angiogenesis process such as CXCR4 and CCL12^44^ (Table S4). In addition, the group that received NSAID during the active phase demonstrated a downregulation of transcripts associated with the expression of pro-inflammatory cytokines including Stat1, IL-9, IL-6ra, and IL-18^48,49^.

Bone healing involves an early inflammatory phase in which immune cells such as neutrophils, natural killer cells (NK) and macrophages, as well as a variety of cytokines, initiate the healing cascade. Cytokine expression varies throughout the post-fracture bone healing period; early stages of bone fracture healing present with inflammatory cytokines that are eventually replaced by anti-inflammatory cytokines^50^. It has been demonstrated that macrophages regulate the recruitment and activation of mesenchymal stem cells by secreting cytokines such as IL-1β, IL-6, and TNF-α during the early period of the inflammatory phase^51^. In the subsequent period, the mesenchymal stem cells immunoregulate the macrophages toward anti-inflammatory phenotypes through a COX-dependent pathway that involves the production of PGE2. Afterwards, M2 macrophages produce anti-inflammatory cytokines such as IL-4, IL-10, and IL-13 along with oncostatin M (OSM) to induce osteogenesis, and thus, inflammation is dampened and the tissue repair process is initiated^51–53^. The resolution of inflammation is a prerequisite for efficient tissue repair^54^. Prolonged inflammatory reactions associated with elevated levels of pro-inflammatory markers appear to delay revascularization and affect the healing outcome^54–58^.

We found signs of a prolonged inflammatory phase in the group that received NSAID at resting time, while there was an expression of anti-inflammatory cytokines (IL-13, IL-4 and angiogenic factor VEGF) and a decreased level of pro-inflammatory mediators and chemokines (IL-1β and IP-10) at day 3 following surgery in the group that received NSAID during the active phase.

The COX enzyme, the key target of NSAID, plays a vital role in the generation of the inflammatory response during the bone healing process by converting arachidonic acid to prostaglandins^9^. Also, the COX enzyme and PGE2 have been found to affect the response of immune cells such as macrophages during bone healing in the synthesis and release of pro-iflammatory and anti-inflammtory mediators^15,59,60^. Our study indicates that administrating NSAID during the active phase could modulate the synthesis and release of these cytokines by immune cells (e.g., macrophages) through the COX-inhibition pathway, decreasing pro-inflammatory cytokines (e.g., IL-1β and interferon gamma-induced protein 10) and increasing anti-inflammatory cytokines (e.g., IL-13 and IL-4) (Fig. 6a,b).

NSAID administration during the active phase after bone fracture improved bone healing by increasing the percentage of mineralization tissue at the fracture site and decreasing the number of osteoclast cells, which resulted in increased bone strength and better postoperative recovery. This effect on bone metabolism was probably a result of COX-dependent and COX-independent inflammatory pathways affected by NSAID chronotherapy.

Results from recent studies showed that endochondral ossification in fracture healing involves the circadian clock genes, especially Per2^21^. Taken together, the differential expression of the Per2 clock gene and the over-representation of the circadian clock pathway along with other inflammation and immune pathways suggested an immune and inflammatory response in the mice that received NSAID during the active phase that may improve bone healing. Also, the microarray gene expression and cytokines analysis suggested that the timing of NSAID administration after bone fracture stimulated the healing process in the group that received NSAID during the active phase.

There is growing evidence indicating an association cytokine serum levels, and circadian clock genes like Per2^12,13^. For example, mice with Per2 mutations present alterations in the serum levels of immune cytokines like IL-1B and VEGF, indicating that Per2 regulates these cytokines^14,43^. Thus on explanation to the changes in immune cytokine levels we observed in our study could be the effects chronotherapy on the Per2 gene.

We observed an earlier resolution of the inflammatory process, which was manifested by an anti-inflammatory state (increased serum levels of IL-13, IL-4 and VEGF) mainly due to the expression of genes that are involved in the polarization of macrophage cells toward anti-inflammatory phenotype M2, which is associated with better healing outcomes^53^. Schmidt and Serhan demonstrated that between the first day and day three after trauma, the upregulated anti-inflammatory signalling coincides with the overexpression of angiogenic factors such as VEGF in the hematoma of the bony environment^54,56^.

The M1 macrophage is associated with bone destruction and M2 cells with tissue repair. M2 macrophage cells produce anti-inflammatory cytokines such as IL-10, IL-13, and IL-4 and promote osteogenic differentiation of bone marrow-derived mesenchymal stem cells, the precursor of the osteoblast^61^. The differentiation of mesenchymal stem cell to osteoblast requires direct cell-cell contact with macrophage cells (M2 phenotype), and the production of a soluble factor known as oncostatin M. Oncostatin M production from this cell-cell contact is regulated by prostaglandin E2 and cyclooxygenase 2 loops^62^.

Our study has several limitations. First, for the pain behaviour and bone healing experiment following NSAID administration we used 2 different surgery times, the group receiving morning NSAID treatment had the surgery done in the morning while the group receiving evening NSAID treatment had the surgery in the evening. This allowed us to fix the time between surgery and NSAID administration in both groups. However, this approach may have introduced a bias, which is the difference in time of surgery between the two groups, and this is a limitation of our study design. Nonetheless, the fact that surgery time did not have a significant effect in our preliminary experiment (without NSAID) provides validity to our findings. Secondly, to assess the effect of NSAID chronotherapy on COX enzyme level, PGE2 and LTA4 we used the RNA gene expression analysis, which revealed that the expression of two genes related to arachidonic acid pathway, the prostaglandin I receptor gene (ptgir) and Arachidonate 5-lipooxygenase activating protein (Alox5ap) gene, were downregulated differently depending on the timing of NSAID administration; downregulated more in the active time group.

Finally, choosing ZT 2 and ZT 13 as timepoints for surgery and NSAID administration was also a limitation in this study. Indeed, using ZT 6 or ZT 18 time points would have allowed us to detect stronger effects of NSAID chronotherapy since these timepoints correspond to the highest peaks and lowest nadirs of inflammatory activity respectively^63^. However, this would have had limited clinical applicability towards the management of bone fractures, and surgeries in humans, as they rarely occur while sleeping in the middle of the night, and patients don’t wake up in the middle of the night to take their medications. Therefore, even though the ZT6-18 strategy would have allowed us to observe more pronounced effects, its translation to clinical implications is limited. Nonetheless our results indicate that the effect of NSAID chronotherapy was strong enough to be detected with ZT 2 and ZT 13 timepoints.

## Methods

### Study design

The primary objective was to estimate the effect of timing NSAID administration on pain and bone fracture healing. In the first experiment we assess the effect of timing of bone fracture surgery in mice, second experiment was to test the effect of NSAID administration during active period compared to resting phase after bone fracture surgery in mice on pain and postoperative recovery. The third experiment was to identify serum cytokines and different gene expression from the healing callus of fractured bone in ice between those animals who received NSAID at different time of the day. For all experiments, replicate numbers are outlined in Materials and Methods or figure legends. Minimum sample size was determined by power analysis based on an estimated formula. Mice in all experiments were age-matched and randomized into groups. Experimenters were blinded to experimental groups to remove bias. Adverse animal welfare issues were sufficient to halt experiments but did not arise during this work.

### The effect of the timing of bone surgery on bone healing and recovery

To assess the effect of the timing of bone fracture on postoperative pain and bone healing, we used a tibia-fracture model in mice. In this model, animals underwent bone fracture surgery at two different times of the day (active and resting time). Behavioural indices of pain including weight-bearing and guarding tests, as well as the biomechanical and histomorphometric properties of the healing bones were examined.

### Animal model

We acquired sixteen 4-months-old wild-type C57BL/6j mice weighing 25 to 28 g from Jackson Laboratories (Bar Harbor, ME). We chose this strain, which is deficient in melatonin, to identify the pathways independent of the melatonin systemic effect^64^. The mice were housed in pathogen-free conditions at 22 °C with a 12-hour alternating light/dark cycle fed on water ad libitum.

### Experimental groups

Cage assignment, mouse numbering and allocation to study groups were performed by a researcher not otherwise involved in the study procedures. Mice were randomly assigned to one of two study groups following an automated process using the Experimental Design Assistant from the National Centre for the Replacement and Reduction of Animals in Research.

One group received open tibia-fracture surgery at the beginning of the resting phase corresponding to zeitgeber time ZT2, where ZT0 refers to the time the lights turn on in the animal facility (6:00 am). The second group had the same surgery at ZT13, which corresponds to the beginning of the active phase when the light is off (from 6:00 pm onwards). Mice are nocturnal animals in which the active phase starts at the beginning of the night and ends in the morning. These time points were selected because they could be easily translated into clinical practice.

Before the surgical intervention, mice were acclimated for two weeks to the environment and the facility and exposed to behaviour testing equipment twice before obtaining baseline measurements.

### Surgical intervention

For our study, we used the open osteotomy fracture model developed by Grestenfield *et al*.^65^. The tibia fracture surgical procedure adopted by previous studies from the lab^66^ and used by other groups at McGill animal facility^67^. All surgeries were performed by the same operator who was not involved in the randomization, allocation concealment process or outcome measurements. Anesthesia was induced initially with a 4% isoflurane/oxygen mixture and maintained at 2%, and a buprenorphine (5%) subcutaneous injection was given before surgery for pain control. An incision centred over the knee joint medial to the patellar ligament was done to create an entry portal to the tibia medullary canal using a 27-gauge × ½ inch TB syringe. The stylet of a 25-gauge spinal needle was inserted in an intergrade fashion through the lumen of the 27-gauge needle down to the distal growth plate, and its tip was used to bend the spinal needle wire, which was cut 1 mm above the bent, then was inserted and adapted smoothly beneath the patellar pliers. The wound was closed with a 5-0 absorbable vicryl suture. Mice were monitored for any signs of improper healing or infection. All animals were euthanized two weeks later at the same time of the day.

### Pain assessment

Behavioural indices of pain including weight bearing and guarding were assessed at baseline (pre-surgery), and on day 7 and 14 after fracture surgery. All assessments were performed at the same time of the day by an investigator blinded to the experimental design and intervention group.

### Guarding behaviour test (GBT)

Guarding behaviour test was done according to already stablished protocol used by Yasuda *et al*.^68^. Each mouse was placed individually on an elevated stainless-steel mesh. Both paws (of the fractured and non-fractured legs) were closely observed for 1-minute periods every five minutes over one hour (12 measurements). A score of 0, 1, or 2 had been given per the postural position of each paw in 1-minute scoring periods. Score 0 was given if the injured side is blanched or distorted by the mesh (indicating weight bearing); and score 2 means that the paw is completely off the floor, score 1 was given if the paw touched the floor without blanching or distorting. After one hour session, the sum of the 12 measurements which could range from score 0 to score 24 during the session was calculated for each paw. Subsequently, the scores of the fractured or non-fractured leg were compared between different experimental groups.

### Weight-bearing test (WBT)

Weight bearing test was done to test the recovery of the treated limbs according to an already published and established protocol^69^. The relative distribution of weight the animal bear on the injured, and uninjured legs was determined by an in-capacitance meter (IITC.inc, CA, US). Each mouse was positioned in an angled chamber so that each hind paw rests on a separate weighting plate. The weight exerted by each hind leg was measured for 5 seconds and then averaged. The change in paw weight distribution was calculated by determining the difference in weight (g) between the left (control) and right (fractured) legs. This difference had been used as a pain index in the fractured leg.

### Assessments of the healing bones

Following euthanasia, the harvested tibiae were examined by microcomputed tomography (µ-CT), biomechanical testing and histomorphometric analyses.

### Microcomputed tomography (μ-CT) assessment

*Ex-vivo* scans of 14-day harvested tibiae were conducted using a micro-CT (Skyscan 1172; Bruker-microCT, Kontich, Belgium), the methodology in this test was based on two previous studies^66,67^. Scans were taken with a 5.88 mm pixel size, at scanner voltage and current set to 59 kV and 167 lA, respectively. 3D reconstructions were created using the (CT-Analyser; Bruker micro-CT, Kontich, Belgium) software to measure the callus size, total volume (TV), and bone volume (BV). The bone volume fraction (BV/TV), and the trabecular number and thickness were calculated for the volume of interest, which encompasses the fracture callus within a 1.5 µm (255 slices) vertical range, centered on the osteotomy site. The region of interest (ROI) for each section was selected as the outer boundary of the fracture callus, excluding the fibula. A binary threshold gray level of 68/255, corresponding to the murine trabecular bone, was used to segment mineralized bone from soft tissue^70–73^ (Table S5).

### Biomechanical testing

The biomechanical properties of the fracture site were tested according to previous studies^67,74^. A three-point bending test performed using a Mach-1a mechanical testing machine (Biomomentum®, Laval, Quebec). The distance between the supports with the bending fixture was 10 mm, and the diameter of the supports and loading nose was 0.25 mm. A downward bending load applied to the middle of the shaft of the posterior aspect of the fractured tibia (over the fractured site) at a rate of 0.016 mm/second until failure. A load-displacement curve generated using Mach-1 software (Tempe, Arizona); this was used to determine three parameters: stiffness (N/mm), ultimate force (N), and work to failure (N*mm).

### Histological analysis

The methodology was previously described in Utvag *et al*.^75^. The bone fracture specimen (right tibiae) were dehydrated in ascending concentrations of ethanol (70–95%) and embedded in methyl methacrylate. After polymerization, three subsequent 6-μm-thick sagittal sections, crossing through the middle of the defect, were obtained from each sample and stained with either tartrate resistance acid phosphatase (TRAP), Masson’s-trichrome or Von-Kossa stain to assess osteoclasts, collagen, and mineralization, respectively. For each biopsy, one histological section per stain was analysed. The histological slides were imaged using an optical microscope (Zeiss-Microscopy, Jena, Germany). Regions of interest for osteoclast, collagen and mineralized tissue histomorphometry were defined as the area of the histological section delimited by the callus and the cortical margins of the bone defects. Osteoclasts were quantified using the ZEN-2012-SP2 imaging software (Zeiss-Microscopy, Jena, Germany) and data was presented as osteoclast number per square millimeter of mineralized tissue (OC/mm^2^). The percentages of mineralized tissue and collagen in the defect analysed using ImageJ v1.45 (Wayne Rasband; NIH, Bethesda, MD, USA) and data presented as mineralized tissue percent (MT%)^76^. Histomorphometric analysis performed by an operator, who was blinded to group allocation.

### The effect of the timing of NSAID administration on pain and bone healing

To assess the effect of the timing of NSAID administration on bone healing and pain, we conducted an experiment using 16 mice. The housing and handling of mice and the surgical intervention were performed as described above.

Mice were randomly assigned to one of two study groups; one group received fracture surgery and postoperative subcutaneous injections of NSAID (carprofen 20 mg/kg) only at resting time (ZT2) for three days, whereas the second group had the same treatment at the active time (ZT13). The carprofen dose is equivalent to a typical postoperative prescription of ibuprofen of 500 mg every 8 hours^77^. Mice were observed for any signs of improper healing or infection. All animals were euthanized two weeks after surgery.

Pain behaviour assessments after bone fracture surgery and the examination of the harvested bones were carried out as described above.

### Identify inflammatory cytokines and metabolic pathways affected by the timing of NSAID administration after three days of bone fracture surgery

To address this aim, we performed a third experiment with nine mice randomly assigned to three groups. One group received an NSAID treatment for three days after bone fracture surgery during resting time (ZT 2) and the second group received the same treatment during active time (ZT13), while a third group did not receive any surgery or NSAID. Surgery was performed for the NSAID groups as described above at the same time of the day (surgery day 0) for all mice. Mice were observed for any signs of improper healing or infection after surgery. All animals were euthanized on day three after the surgery at 12 pm (ZT 6). This was 78 hours after the surgery; >30 hours after the final NSAID dose in the “resting time” group and >18 hours in the “active time” group. This is enough time to eliminate the drug from the system and rule out possible confounding effects due to the presence of the drug in the blood, as the half-life of carprofen in rats is 8 hours^78^.

### Assessment of early healing at the molecular level

We performed a serum cytokine analysis and RNA microarray sequencing of the healing callus three days after surgery.

### Cytokines profile for blood serum

Blood serum was collected from each mouse at day three after the surgery and frozen at −80° in separate vials until further analysis. For cytokine analysis, the serum samples were thawed at room temperature. A multiplex inflammatory cytokine kit (Mouse Cytokine Magnetic 20-Plex Panel-Thermofisher Scientific, Waltham, MA) and the Luminex 100/200 Multiplexing Instrument (Luminex Corp., Austin, TX) was used to analyze the inflammatory cytokine profile of each serum sample. The Mouse Cytokine Magnetic 20-Plex kit was used for bead assays of FGF basic, GM-CSF, IFN-γ, IL-1α, IL-1β, IL-2, IL-4, IL-5, IL-6, IL-10, IL-12 (p40/p70), IL-13, IL-17, IP-10, KC, MCP-1, MIG, MIP-1α, TNF-α, and VEGF. These assays were performed following the manufacturer’s protocol. The Luminex®100/200 System and xPONENT instrumentation software were used for measurements and analysis. All experiments were performed in triplicates, and the mean fluorescence intensity (MFI) for each cytokine (the raw data) was used to calculate the concentration of each cytokine^79^.

### RNA isolation and whole-transcript expression analysis

The central one-third of the fractured tibia including all the callous and hematoma was harvested on day three post-fracture from the experimental groups and stored in Alloprotect (Qiagen, Toronto, Ontario). The total RNA was isolated from bone tissue using QAlzol (Qiagen, Toronto, Ontario) and then purified using RNeasy Lipid Tissue Mini Kit columns (Qiagen, Toronto, Ontario) following the manufacturer’s instructions. The extracted total RNA sent for Whole-Transcript Expression Analysis. Total RNA was quantified using a NanoDrop Spectrophotometer ND-1000 (NanoDrop Technologies, Inc., Waltham, MA) and its integrity was assessed using a 2100 Bioanalyzer (Agilent Technologies, Waltham, MA) (Fig. S3a,b). Sense-strand cDNA synthesized from 100 ng of total RNA, and fragmentation and labeling were performed to produce ssDNA with the Affymetrix GeneChip® WT Terminal Labeling Kit (Thermofisher, Waltham, MA) according to the manufacturer’s instructions. After fragmentation and labeling, 3.5 µg DNA target was hybridized on GeneChip® Mouse Gene 2.0 ST (Affymetrix, Waltham, MA) and was incubated at 450 C in the Genechip® Hybridization oven 640 (Affymetrix, Waltham, MA) for 17 hours at 60 rpm. The GeneChips then were washed in a GeneChips® Fluidics Station 450 (Affymetrix) using Affymetrix Hybridization Wash and Stain kit according to the manufacturer’s instructions (Affymetrix). The microarrays finally were scanned on a GeneChip® scanner 3000 (Affymetrix). The data was normalized and only the entities that were flagged as being present in the samples included in the analysis (GeneSpring GX 10, Agilent). To identify differences in gene expression the three experimental groups: unfracture control, gorup of mice that received NSAID during active phase after bone fracture, and gorup that received NSAID at resting phase; we computed unpaired student’s t-test between corresponding groups. Differences in gene expression with p values less than 0.05 that demonstrated an up- or down-regulation by more than 1.5-fold were identified.

Using Go Ontology database (PANTHER Classification version 11)^80^, PANTHER Overrepresentation Test was performed on the list of genes that were expressed significantly differently (p < 0.05) to identify the functional biological processes involved by these genes. The proportional distribution of genes in each process was calculated.

The list of genes that significantly differentiated between activity phase group to resting phase was employed to identify significantly activated pathways by comparing their functional annotations according to the PANTHER classification systems (PANTHER overrepresentation test, Reactome Pathways, version 11(2016), www.pantherdb.org) with the whole mouse genome (data updated to NCBI’s January 10 2011, release)^80^.

### Statistics

The sample size for the groups of the first two experiments was calculated on o.8 power, based on a coefficient of variation of 25% in the types of data that are collected, and after accepting α and β errors of 5%. All data expressed as mean ± SE. The normal distribution of the data was checked using Kolmogorov-Smirnov test. For comparison between two groups, unpaired Student’s t-tests used when the data was normally distributed if not Mann-Whitney test was performed. Two-way ANOVA analysis followed by Fisher LSD post hoc test used for comparison between groups at different time points. Statistical analyses were performed using OriginPro 2017 (OriginLab, Northampton, MA). Differences were considered significant at p < 0.05.

For the cytokines experiment, a sample size of 3 with three replicated was used to achieve an α = 0.05 and a power of o.8 according to previous studies^81,82^. Data not normally distributed were analysed using Mann-Whitney test. OriginPro 2017 (OriginLab, Northampton, MA). For gene expression data, the PANTHER classification systems (PANTHER overrepresentation test, Reactome Pathways, version 11(2016), www.pantherdb.org) with the whole mouse genome (data updated to NCBI’s January 10 2011, release)^83^. Differences were “considered significant at p < 0.05”^84–103^.

### Study approval

Study approval was obtained from the Animal Care Committee at McGill University. We confirm all experiments were performed in accordance with relevant guidelines and regulations.

## Supplementary information

Supplementary information Appendix.
