# Supplementary material for: Chronotherapy of Non-Steroidal Anti-Inflammatory Drugs May Enhance Postoperative Recovery
Source: Sci Rep. 2020 Jan 16;10:468. doi: 10.1038/s41598-019-57215-y (PMC6965200; doi:10.1038/s41598-019-57215-y)
Supplement: Supplementary file 1 — Supplementary information Appendix. [file 41598_2019_57215_MOESM1_ESM.docx]

**Supplementary information Appendix**

**Chronotherapy of Non-Steroidal Anti-Inflammatory Drugs may Enhance Postoperative Recovery**

Al-Waeli H.^1^, Nicolau B.^1^, Stone L.^2^, Abu Nada L.^2^, Gao Q.^2^, Abdullah M.N.^3^, Abdulkader E.^1^ Miyako Suzuki ^2^ , Mansour A.^2^, Al Subaie A.^2^, and Tamimi F^2^.

1. Faculty of Dentistry, McGill University, 2001 Avenue McGill College Suite 500, Montréal, QC, H3A 1G1, Canada.
2. Faculty of Dentistry, McGill University, Strathcona Anatomy and Dentistry Building, Montreal, QC H3A 0C7, Canada.
3. Faculty of Dentistry, University of Toronto, 124 Edward St, Toronto, Ontario, M5S, Canada.

Corresponding author:

Faleh Tamimi, Faculty of Dentistry, McGill University, Strathcona Anatomy and Dentistry Building, Montreal, QC H3A 0C7, Canada.

E mail: f.tamimimarino@mcgill.ca

**Table S1.** **Phases during fracture healing in mice**

**Timing of cellular and expression of signalling molecules during mice fracture healing ^44,52,56,84,85^.**

| Day | Event | Molecular signalling | |
| --- | --- | --- | --- |
|  |  | **Cells** | **Signals/Cytokine** |
| 1 | **Hematoma formation and initiation of acute inflammation**  (Activation and recruitment of proinflammatory cells and cytokines) | Macrophages, neutrophils and attraction of mesenchymal stem cells | ***IL-1, IL-6, IL-8, IL-11, TNF-α, CCL2, CCL7, IP-10*** (from inflammatory cells-Monocytes /macrophage)  ***PDGF, TFG-β*** (from degranulation platelets)  ***BMP-2*** expression  ***GDF-8*** and osteoprotegerin expression |
| 3 | **Fibrous tissue**  (Angiogenesis) | Mesenchymal stem cells, osteoblast start to differentiate in intramembranous bone | Expression of: ***CCL3***, ***CCL11, IL-10, HIF1 α protein, HMOX, VEGF and GLUT1, TGF-β2, β3, GDF-10, BMP-5, -6 and RANKL and M-CSF, MIF (decreased for 2wks)***  Decline of cytokines: ***CCL7, IL-6, IL-1***  Induction of ***Angiopoietin-1*** |
| 7 | **Soft and hard callus formation** (Endochondral ossification) | Intramembranous ossification’s cell proliferation (peaks between 7-9 days). Maturation of chondrocytes (days 9-14) | Expression of ***CXCL12***  The peak of ***TGF-*** ***β2, β3 &*** osteoprotegerin  Expression of ***GDF-5 and*** probably ***GDF-1*** |
| 14 | **Cartilage resorption and Active osteogenesis** | Osteoblastic and osteoclastic cells activities, along with new mesenchymal cells | Decreased levels of expression for ***TGF-b2, GDF-5, and*** probably ***GDF-1***  Expression of ***BMP-3, -4, -7, and -8***  Expression of ***VEGF and RANKL, M-CSF***  Peak of ***IL-1 and TNF-a*** expression that continues during bone remodelling |
| 21 | **Remodelling** | Continuous osteoblastic and osteoclastic activities | Second peak of ***IL-1, IL-6, and TNF-a*** expression which continues during bone remodelling |

**Table S2. Circadian rhythm of immune cells and cytokines expression during the day in various species (Human and Mice).**

| **Cytokines/ Chemokines ligands or receptors/** | **Role in inflammation, and bone healing** | **Circadian Rhythm** | **Reference** |
| --- | --- | --- | --- |
| **IL-4** | Th2 cytokines that are associated with macrophage M1-M2 polarization. Expresses at 3-4 days after fracture associated with anti-inflammatory macrophage trait | H-ZT18 | ^86^  ^44^ |
| **VEGF** | Secreted by endothelial cells, induce angiogenesis and growth. Expresses at the end of hematoma formation when the hypoxia state start (around 4 to 14 days after fracture) | M-serum  Peak during the dark phase and lower levels at light phase (ZT2 and ZT14) | ^17^ |
| **IL-6** | IL-6 is the cytokine largely responsible for inducing the synthesis of the acute phase proteins C reactive protein and serum amyloid protein A (SAA) and is one of  the major cytokines involved in bone resorption. Highly expressed in day 1 and with presence of inflammation expressed at day 7 and 14 | H-Morning 7:30  Healthy and RA patients at 4:00 am | ^87^, ^88^  ^85^ |
| **IL-10** | Healing in fetus^89^, Expressed at day 4-7 after fracture | H-Morning 7:30 with second peak at 13:30 | ^87^ |
| **TNF-a**  **Moreover, its receptors (p55 and p66)** | expressed in day 1 and later in day 21 and 28, the depressed level at day 7 | H-S-Morning 7:30  6:00 am in healthy and RA patients | ^87^  ^85^ |
| **MMP-9** | Potent degenerative enzyme | H-Tears-Increase 200-fold on awakens | ^90^ |
| **IL-1B** | One of the most potent osteoclast-activating factors within the human organism and is thus believed to play  an important role in periodontal tissue breakdown, IL-1 peak ion the 3rd day after fracture, expressed in day 1 and later in day 21 and 28, depressed level at day 7 | H-GCF-  Periodontal healthy subjects-lowest morning, highest evening (melatonin can affect) | ^91^  ^92^ |
| **Osteoprogetron (OPG) and its receptor (RNKL)** | Peaks in the fracture site after 24hours and at the peak of cartilage formation phase day 7, while RANKL osteoprotegerin was seen on day 3 and 14 like MCSF |  | ^85^ |
| **IL-1R1 and receptors** | Expresse on day 1 and 3 post fracture |  | ^85^ |
| **IL-5** | Recruitment and activation of the OPG and inhibition of osteoclast activity |  | ^93^ |
| **Macrophage inhibitory factor (MIF)** | Expressed at day 4 of fracture and decreased gradually for two weeks.  Counter-regulator of glucocorticoid action.  Induced immunosuppression and glucocorticoid  Induced proinflammatory cytokine inhibition.  8% of macrophage cells and their secretion is under local circadian control-IL-6 or TNF-a | H- peaks 6:00-9:00 am  Nadir: 00:00 to 3:00 | ^94^, ^95^  ^96^  ^15^ |
| **CXchemokines ligands**  **CXCL12** | Induced recruitment and retention of haemopoietic stem cells and macrophage cells and mature immune cells, Upregulate during the acute phase of bone healing | Down regulate at the beginning of the resting phase to allow release of the cells in the blood (ZT23, ZT1, ZT24) | ^44^ |
| **CXCR4** | Receptor of the CCL12 on the cells and CD4 and CD8 and T cells. Associated with the recruitment of BM-MSCs to the fracture site | Peak during the resting phase | ^44^ |
| **P-selectin, E-selectin, VCAM-1 ICAM-1, Ccl2** | Initiation of the inflammatory phase of the endochondral ossification healing. Express in day1 – day3 | M-bone marrow –(ZT13) | ^44^ |
| **INF** | Role in initiation and activation of macrophage polarization from M0 to M1. Expresses in day 3 | Natural killer -M – (ZT 14-24) | ^44^ |
| **TLR9**  **Toll-like receptor 9** | Modulates the inflammatory response during bone helaing and may affect the osteoclastogenesis through its affect on osteoclasts cells or osteoblasts. Its subunit of TLR family | M-ZT19-7 | ^97^ |
| **Immune cells** | **Role in inflammation, and bone healing** | **Circadian Rhythm** | **Reference** |
| **Neutrophils, lymphocyte, monocyte, eosinophils** | Recruited to the fracture site for secretion and initiation the inflammatory phase,  Highly expressed at day 1 | M-CT17 (during active phase) | ^98^ |
| **Macrophages** | Recruited at the beginning of the inflammatory phase at the fracture site (role in secretion of different proinflammatory and anti-inflammatory cytokines) | M-CT0-12  (during resting phase) | ^15^ |
| **Β-cells and T-cells** | During later stage of inflammatory phase, B-cells and T-cells are involved in suppression of the pro-inflammatory signals, and able to induce anti-inflammatory functions from mesenchymal stem cells and has been shown to induce osteogenic differentiation and activity | M-ZT5-13 | ^99^ |

H-Human, M-mouse, CT: actual circadian time in hours (e.g. CT6 = 6AM); ZT: Zeitgeber time: time after the onset of light with lights on at ZT0/24 and off at ZT12ZT 0-light on, ZT12-Light off

**Table S3. The percentage distribution of biological process ontologies identified for upregulated and downregulated between active time administration of NSAID group versus resting time administration of NSAID after fracture surgery on day 3.**

| **Biological processes ontologies** | **N genes (%)**  **Significantly upregulated genes** | **N genes (%)**  **Significantly downregulated genes** |
| --- | --- | --- |
| **Biological regulation** | 5(9.6) | 28(25.5) |
| **Cellular component organization and biogenesis** | 5(9.6) | 19(17.3) |
| **Biological adhesion** | - | 5(4.5) |
| **Cellular process** | 18(34.6) | 47(42.7) |
| **Developmental process** | 1(1.9) | 7(6.4) |
| **Immune system process** | 1(1.9) | 21(19.1) |
| **Localization** | 5(9.6) | 24(21.8) |
| **Locomotion** | 1(1.9) | 2(1.8) |
| **Metabolic process** | 17(32.7) | 42(38.2) |
| **Multicellular organismal process** | 5(9.6) | 18(16.4) |
| **Reproduction** | 1(1.9) | - |
| **Response to stimulus** | 5(9.6) | 25(22.7) |
|  | **64** | **238** |

**statistically significant genes at least 1.5-fold change (p<0.05), N gene hits against total number of genes**


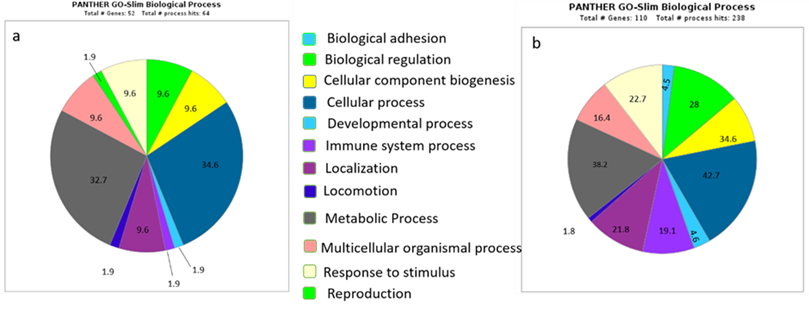


**Fig.S1 The percentage distribution of biological process ontologies identified for statistically significant genes (p < 0.05) differentially upregulated (a) and downregulated (b) between group received NSAID upon active time as compared to that in the group received NSAID at a resting time at day three post-surgery.**

**Table S4. Examples of expressed genes of RNA sequencing analysis in the healing callus. Genes of known function in bone healing after 3 days of NSAID administration at active time in comparison to resting time, p-value < 0.05.**

| **Gene** | **Cells expresses the gen** | **Role in bone healing** | **Reference** |
| --- | --- | --- | --- |
| **Examples of upregulated genes** | | | |
| **CCL3**  **(Chemokine (C-C motif) ligand 3)**  **Macrophage inflammatory protein** | Macrophage | Contribute in the recruitment of mesenchymal stem cells and their differentiation | ^44^ |
| **CCL12**  **(Chemokine (C-C motif) ligand 12)**  **monocyte chemotactic protein** | Macrophage | Contributes in the recruitment of mesenchymal stem cells and their differentiation | ^44^  ^47^ |
| **FGFbp**  **Fibroblast growth factor-binding protein** | Fibroblast | Important in the endothelial cellular proliferation |  |
| **Retnla/FIZZ1**  **Resistin-like molecule alpha** | Macrophage | Polarization from M1 to M2 phenotype | ^47^ |
| **IL-4R1/t6N**  **Interleukine-4 receptor subunit-1** | Macrophage | Important in activation of M1 to M2 polarization | ^47^ |
| **Lzic** | bone marrow mesenchymal stem cell | Accelerates the degradation of phospho-β-catenin, resulting in an increased level of WNT signaling, a recognized pathway in osteogenesis | ^100^ |
| **Examples of downregulated genes** | | | |
| **IL-18**  **Interleukin-18** | Macrophage | Overproduction of IL-18 stimulates IFN-γ production and suppresses IL-4 in vivo, resulting in cortical thinning and decreased bone volume in a mouse model | ^48^ |
| **IL-6rα**  **Interleukin-6 receptor subunit α** | Leukocytes  (Neutrophils- Pain receptor) | Blockade of IL-6 ra decrease pain and improve improves compromised fracture healing | ^101^ |
| **STAT1**  **Signal transducer and activator of transcription 1** | Transcription factor that mediate IFNY, IL-6 and IL-12 and many pro inflammatories after acute insult (like a fracture). | Inhibition of Stat1 showed to accelerate bone healing, STAT1 as a crucial negative regulator for both osteoblast differentiation and osteoclastogenesis, and also suggest that inhibition of STAT1 activity may be beneficial for the treatment of skeletal fracture | ^49^ |
| **IL-9**  **Interleukine-9** | Macrophage and T helper cells | Increased level of IL-9 in the fracture haematoma of delayed bone healing |  |
| **Ostf1**  **Osteoclast stimulating factor** | Osteoclast | Induce bone resorption, and in Knock/out mice there is an increase in trabecular bone mass in long bones | ^102^ |
| **Ptgir**  **EP1**  **Prostaglandin 1 receptor** | Osteoblast | In mouse knockout of this receptor there was an increase in the bone healing. Inhibition of EP1 signalling is a potential means to enhance fracture healing | ^9^  ^103^ |
| **MAPK**  **Mitogen-activated protein kinases** | Family of kinases connects extracellular stimuli with diverse cellular responses ranging from activation or suppression of gene expression to the regulation of cell mortality, growth, and differentiation. | Intra-cellular signal transduction proteins Mediates the cellular response after trauma especially proinflammtory like, IL-6, IL-9 and COX-2, MAPK is  critically involved in osteoclast differentiation of the  mouse bone marrow macrophages | ^104^ |


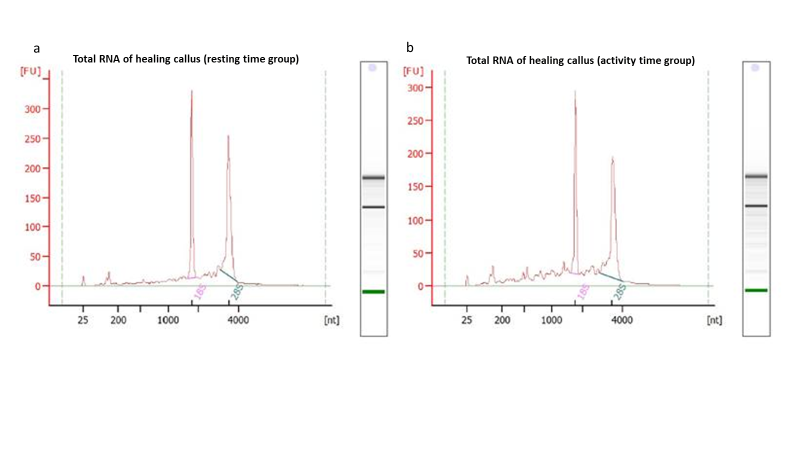
**Fig.S2 Representative Bioanalyzer electropherogram profile of the RNAs contained in the healing callus after open tibia fracture surgery and three days of administration NSAID either during the resting time (a) or during activity time (b). The electrogram show the size distribution in nucleotides (nt) and fluorescent intensity (FU) of total RNA. The most dominant peaks are 18S and 28S. Associated gel image shown alongside the plot.**

**Fig. S3.** **Gene expression heat map for the top 100 genes that showed at least 1.5-fold difference (P<0.05) upregulated (red) or downregulated (blue) at day three after fracture surgery between group receiving NSAID at activity time compared to control (a) and those receiving NSAID at resting time compared to control (b). Gene expression profile of bone healing at day three after fracture surgery between group received NSAID upon active time as compared to that in group received NSAID at resting time. (c) Comparison of common and distinct genes of fractured healing callus after NSAID administration for three days either during activity time or at resting time. Fold-change (FC) vs. FC plot of activity time vs. control (without surgery) on the x-axis and resting time vs. control on the y-axis highlighting common significantly expressed genes (adj.p value p<0.05) in both activity and resting in comparison to control in red, and significantly expressed genes (adj. p<0.05) either during activity or resting time.**


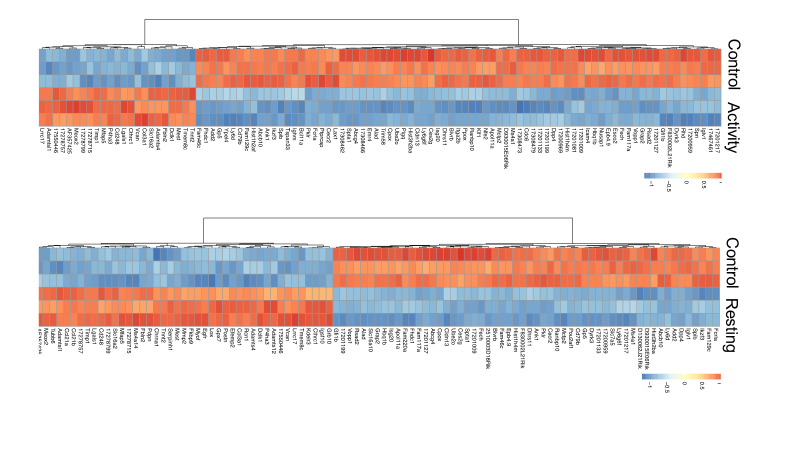


b

a

b


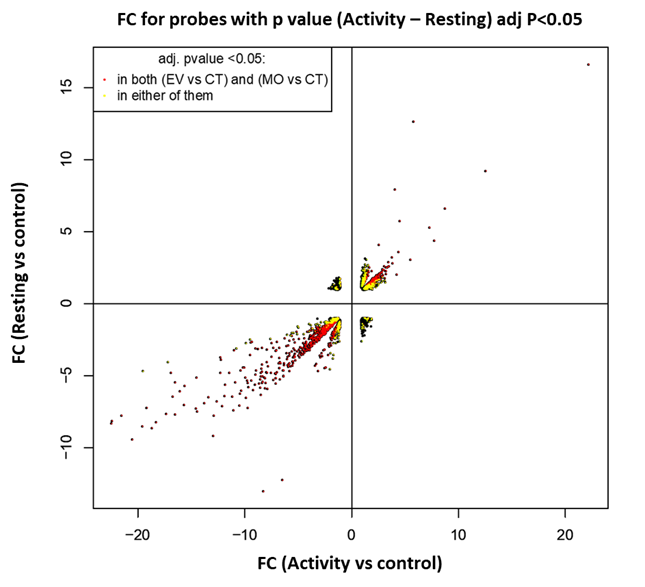


c

**Table S5. µ-CT 3D Reconstruction**

| 1. Open CTAn software(CT-Analyser; Bruker micro-CT, Kontich, Belgium). 2. Click folder button, load reconstructed CT data. 3. On Raw Image, Select the top and bottom section (from the fracture center, approximately ±2mm, end of bone callus).   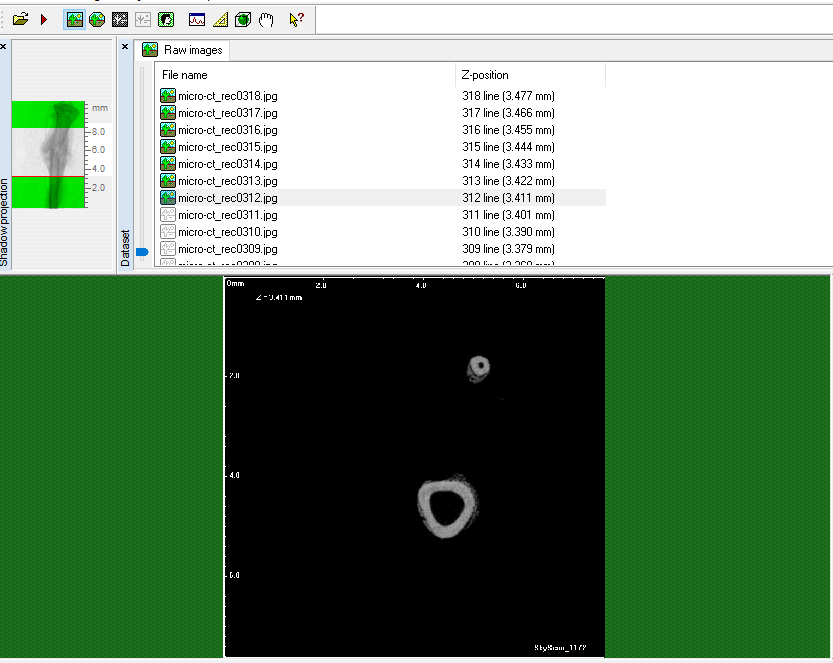 |
| --- |
| 1. Select the biggest callus area, click on <Region of Interest (ROI, and choose all callus area   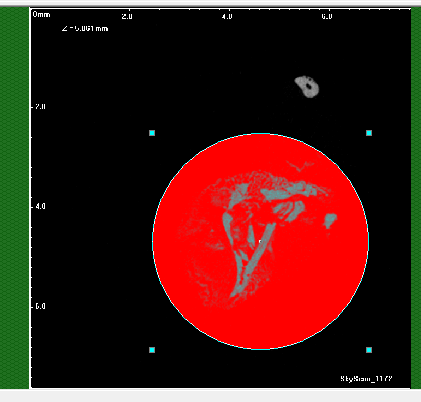 |
| 1. 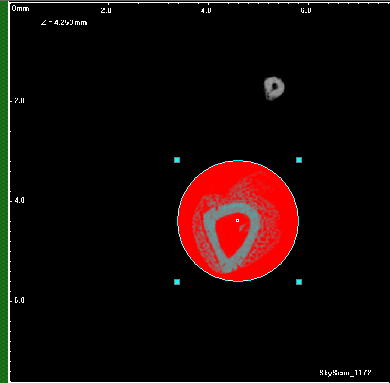Check on other section, whether all the callus is included within ROI. Refine the ROI when move up or down to all the section. |
| 6- Binary selection; to remove all the cortical bone part in the callus tissue  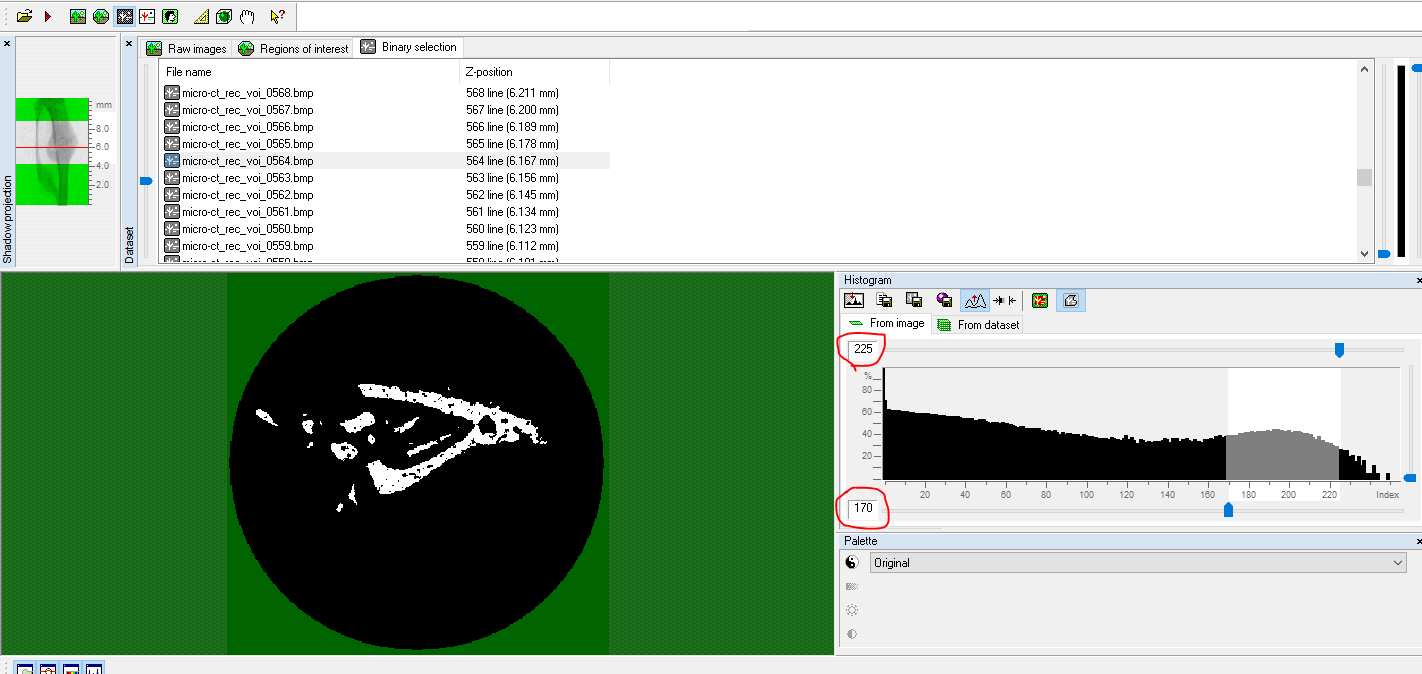 |
| 7- custom processing, then we will see the cortical bone ROI image is overlap with our current ROI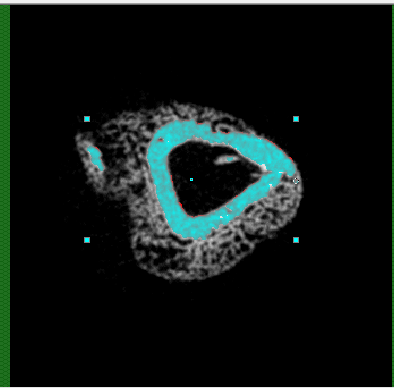  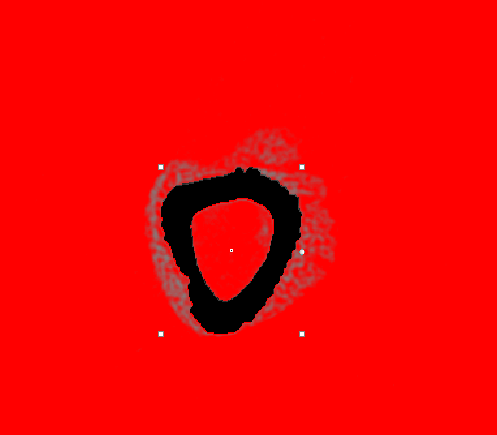 |
| 8- Binary selection, and this time all the cortical bone are removed as the picture below.  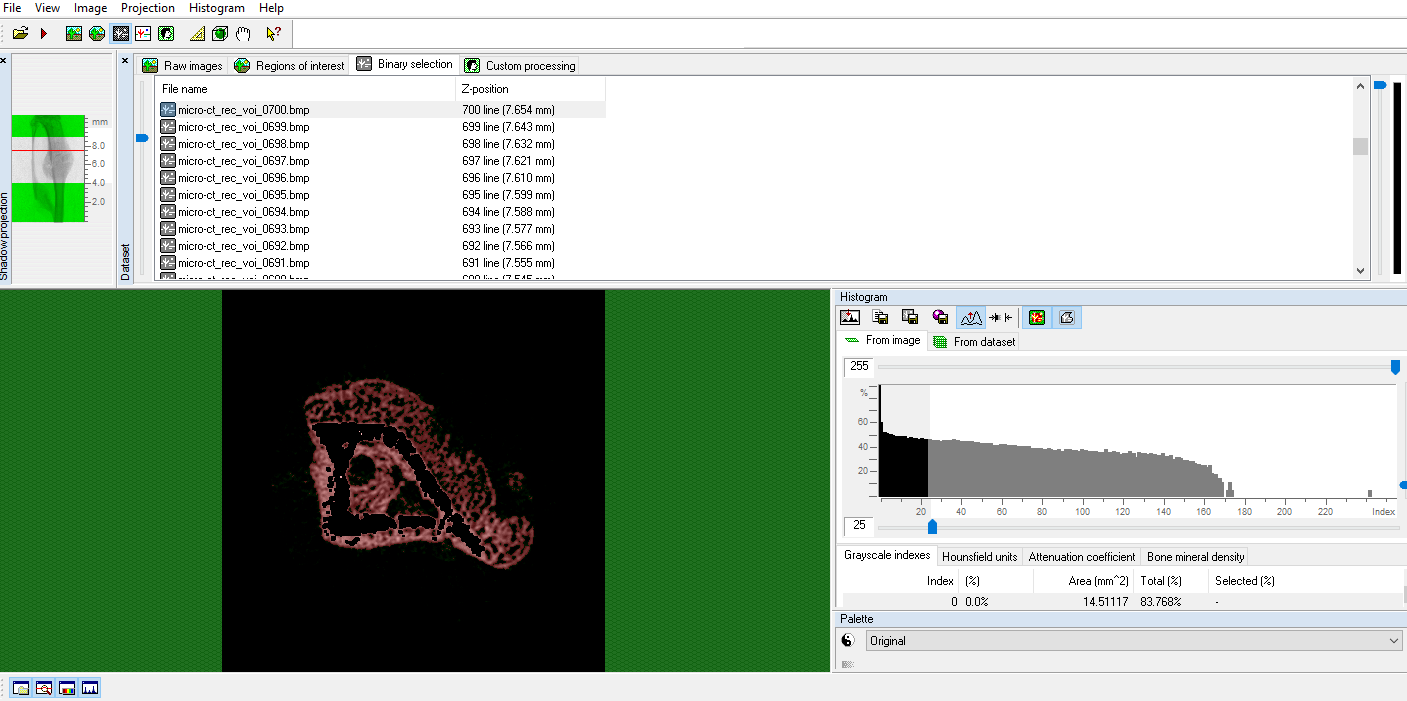 |
| 9- Save and reopen the binary data.  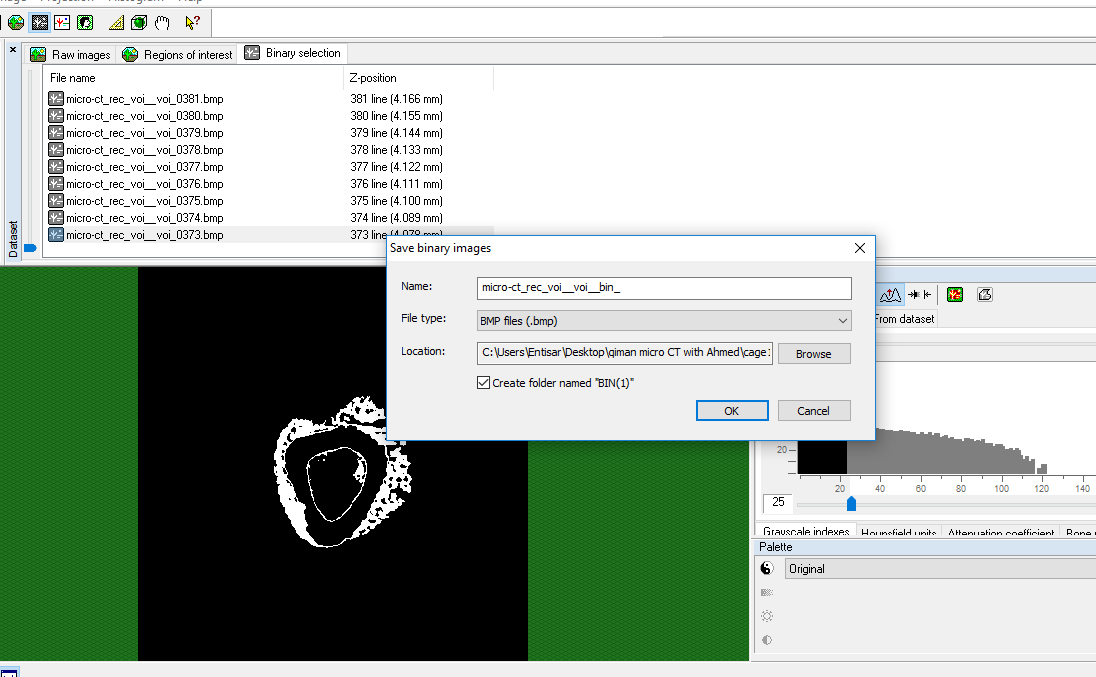 |
| 10- Custom processing, open the internal tab and choose 3D analysis for BV/TV, Trabecular bone number , thickness and spacing. |

**Supplementary pictures**

**
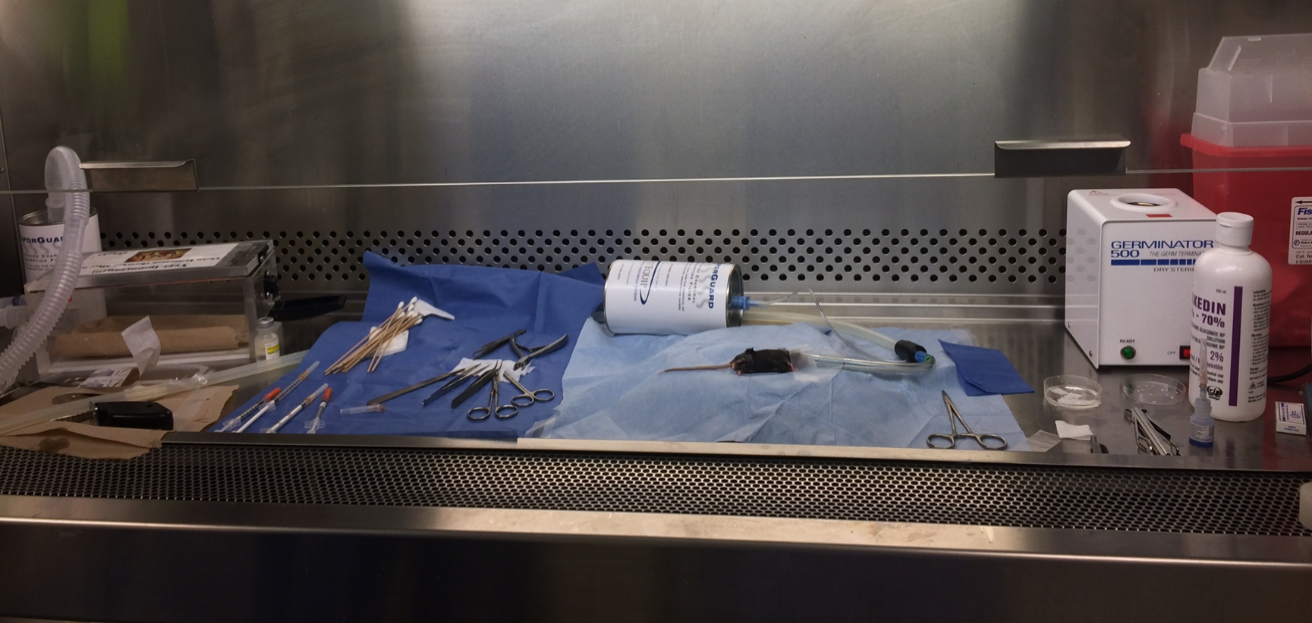
**

Picture S1. Surgical preparation

a

b

c

**
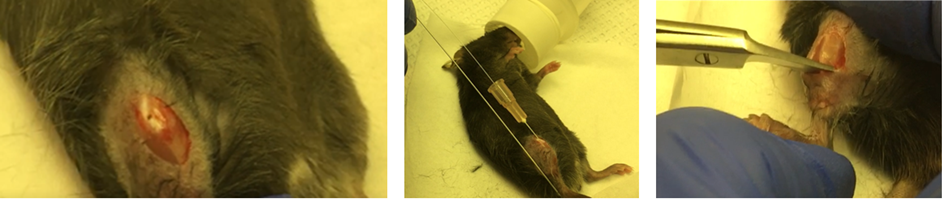
**

Picture S2. Pictures of the open tibia fractured surgery, a) skin incision over the patellar ligament (white tissue), b) insertion of the both the gauge needle followed by the spinal need for internal fixation, c) snap and sharp fracture of the tibia bone.

**
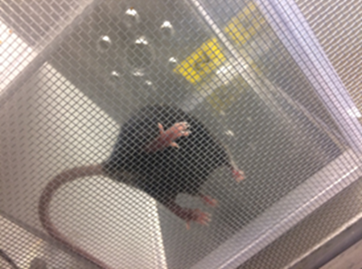

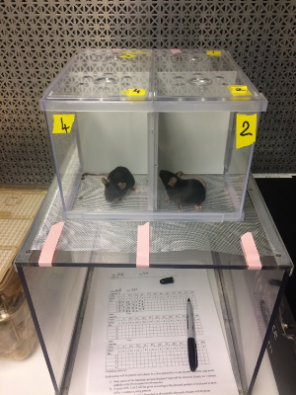
**

Picture S3. Picture of the guarding test mesh for the paw of the fractured leg.

**
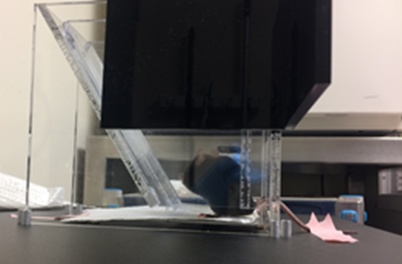

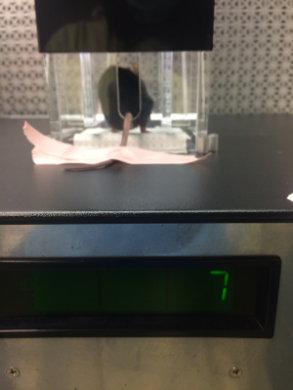
**

Picture S4. Picture of In-capacitance meter chamber with the mouse inside to measure the weight bearing of both legs.


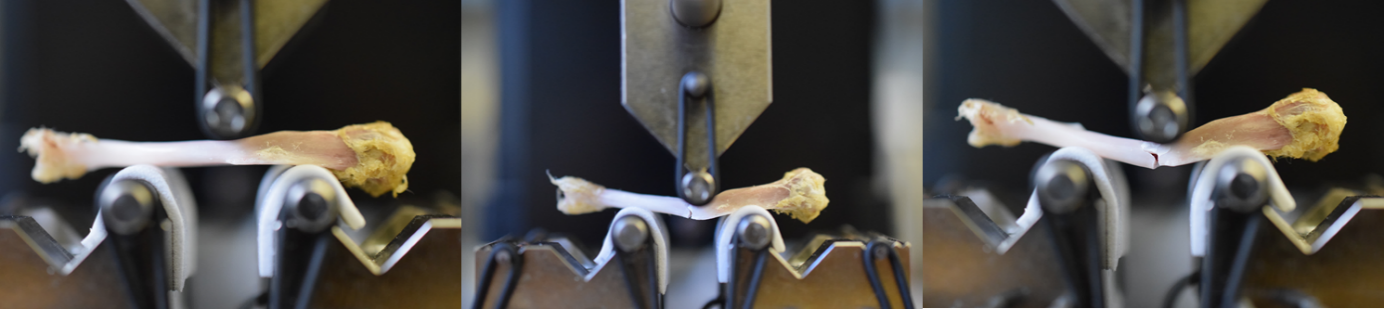


Picture S5. Three points mechanical bending using Mach-1 machine
